# Hemostasis and Beyond: The Diverse Functions of Protein S in Controlling Coagulation, Inflammation, and Cancer

**DOI:** 10.3390/ijms27219932

**Published:** 2026-11-06

**Authors:** Narges Mehrpour-Karmakan, Mélissa E. Jauquet, Jeremy P. Wood

**Affiliations:** Department of Pharmaceutical Sciences, Texas A&M University, Kingsville, TX 78363, USAmelissa.jauquet@tamu.edu (M.E.J.)

**Keywords:** protein S, coagulation, inflammation, cancer, anticoagulation

## Abstract

Protein S (ProS) is a vitamin K-dependent glycoprotein that was first described as an anticoagulant cofactor for activated protein C (APC). Since its initial discovery, studies of ProS have revealed an array of functions, spanning coagulation and inflammation, including in the context of cancer. ProS is now known to inhibit all three coagulation complexes—through interactions with APC, tissue factor pathway inhibitor, and activated factor IX—and to have its anticoagulant activity modulated by multiple other plasma proteins. Beyond hemostasis, ProS also participates in complement regulation, through its interaction with complement component C4b-binding protein, and regulates inflammatory cell phenotype and function, through Tyro3 and Mer-Tyrosine Kinase, receptors which mediate efferocytosis, inflammatory resolution, and tissue repair. Emerging evidence further implicates ProS in cancer biology, where its effects are context-dependent and may either support or suppress tumor progression. In this review, we highlight these many functions of ProS and discuss their mechanistic commonalities. Collectively, these functions position ProS as a central regulatory node, with critical roles in physiology and disease.

## 1. Introduction

Protein S (ProS) was first identified in 1977 during the purification of Protein C from bovine plasma, when an unexpected γ-carboxyglutamic acid (Gla)-containing protein was isolated [1]. As described in a footnote from Di Scipio et al. [1], ProS was originally named after the city of Seattle, Washington, where it was discovered. At the time, nothing was known about the function of this newly discovered protein, and the authors left open the possibility that new information could lead to a future name change. As Protein C was a well-recognized anticoagulant, initial studies to discern ProS activity similarly focused on its role in promoting the inhibitory function of activated protein C (APC) [2,3,4]. However, ProS is now recognized as a multifunctional protein, with diverse roles within the coagulation system, which also extend well beyond hemostasis. These additional functions include managing inflammatory responses through interactions with complement component C4b-binding protein (C4b-BP) [5,6] and macrophage receptors (reviewed in [7]).

In this review, we will discuss the functions of ProS across coagulation, inflammation, and cancer biology, with some emphasis on a model of ProS as an extracellular scaffold. Through its interactions with complement regulators, coagulation factors, and TAM receptors, ProS serves as a central regulatory node in thrombo-inflammation, whose structural and functional versatility shapes diverse physiological and pathological processes.

## 2. Synthesis and Structure of Protein S

**Figure 1:**
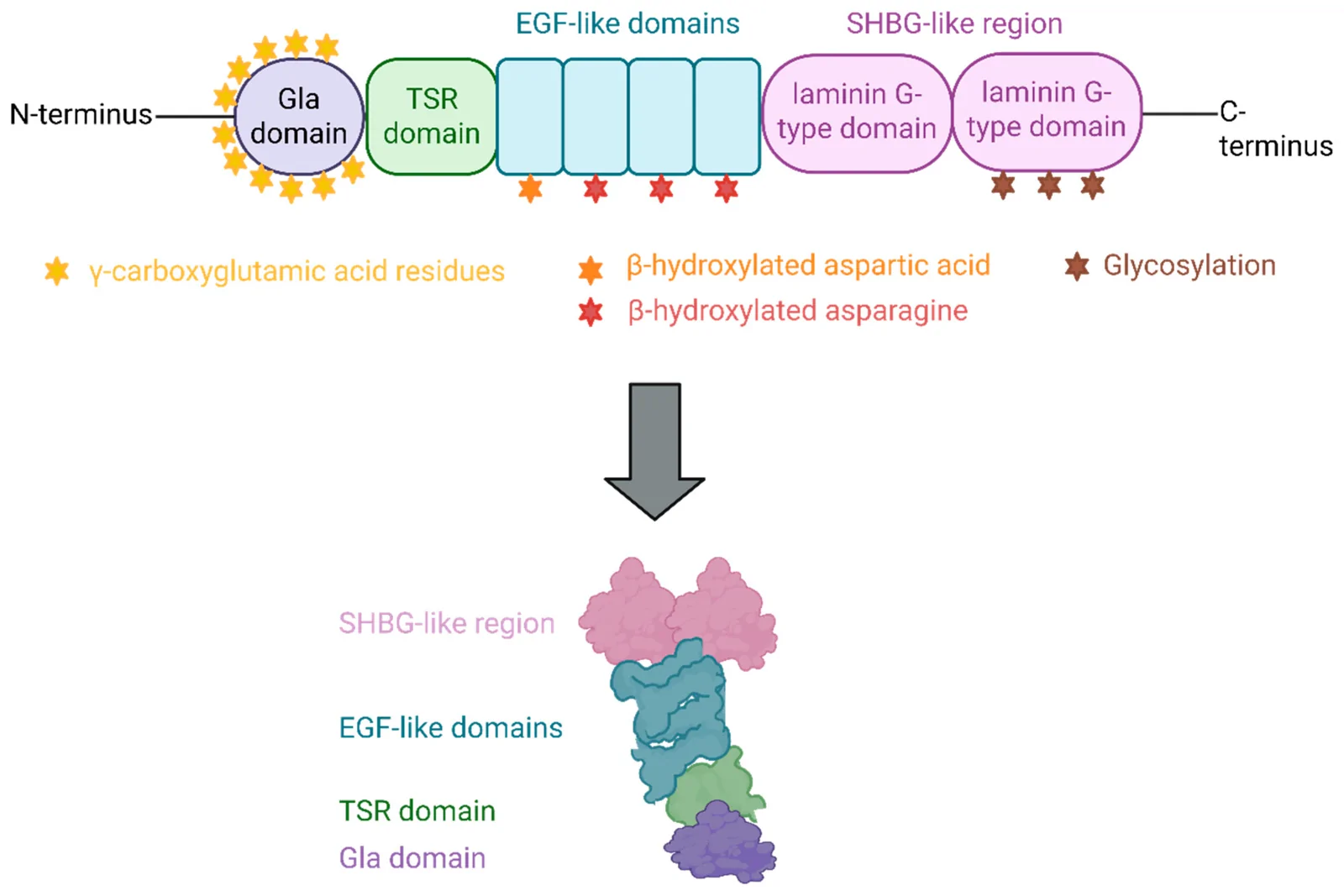
Schematic model of the molecular structure of Protein S (ProS). ProS is composed of 8 domains: the Gla domain, the TSR domain, 4 EGF-like domains, and 2 laminin G-type domains forming the SHBG-like region. Gla: γ-carboxyglutamate; TSR: thrombin-sensitive region; EGF: epidermal growth factor; SHBG: C-terminal sex hormone-binding globulin.

The ProS gene, *PROS1*, is located on chromosome 3q11.2. This gene codes for a precursor protein of 676 amino acids that is processed to the mature ProS of 635 amino acids (approximately 69 kDa) [8,9,10,11]. Essential post-translational modifications are required to obtain a fully functional ProS, including 11 Gla residues in the Gla domain (requiring vitamin K), a β-hydroxylated aspartic acid in the first epidermal growth factor (EGF)-like domain, and a β-hydroxylated asparagine in each of the other three EGF-like domains (Figure 1) [12,13]. There are mRNA forms of ProS, which differ in their 5′ regions and appear to arise from alternative splicing [10].

### 2.1. Protein S Synthesis

ProS circulates in plasma at concentrations ranging from 16 to 30 µg/mL (approximately 210–430 nM), and the concentration varies based on age, sex, and race [14,15,16]. ProS is mainly synthesized in hepatocytes, making the liver the major source of circulating ProS [13,17]. Interestingly, in hepatocytes, ProS can be synthesized and co-secreted along with C4b-BP [18]. C4b-BP-bound ProS represents approximately 60% of the total plasma ProS pool and has reduced anticoagulant activity [19], as described in more detail in Section 3 of this review. The remaining 40% is the free and anticoagulant-active ProS [19]. The second major source of circulating ProS is endothelial cells. Other cells, including macrophages, vascular smooth muscle cells, osteoblasts, neural tumor cells, and Leydig cells in the testis, have also been shown to produce ProS, although the extent to which these cells contribute to the circulating ProS pool remains unclear [13,17,20,21,22,23]. Finally, megakaryocytes also synthesize ProS and package it into alpha-granules of the platelets that they produce. This stored ProS is subsequently secreted upon platelet activation. While it only represents ~2.5% of the total blood ProS, this platelet pool has been shown to be important for preventing venous thrombosis in murine models [8,24].

### 2.2. Protein S Structure

ProS precursor includes an N-terminal signal peptide and propeptide, which are removed during processing [10]. Mature ProS is composed of eight distinct structural domains (Figure 1). At the N-terminal region, the Gla domain, located between amino acid residues 1 and 46 [10], mediates the binding of ProS to negatively charged phospholipid membranes. This interaction is facilitated by multiple calcium ions coordinated within the Gla domain, which promotes membrane association through electrostatic interactions [12,25,26]. Downstream of the Gla domain, there is the disulfide-bridged loop called the thrombin-sensitive region (TSR), which is located between amino acids 47 and 75 [10]. This region contains cleavage sites for thrombin [27] and factor Xa (FXa) [28]. The TSR domain also stabilizes binding to phospholipid membranes and increases the stability of ProS [29]. Following the TSR, four EGF-like domains are located between amino acid residues 76 and 242 [10]. These domains contain high-affinity calcium-binding sites that are critical for the structural stability of ProS and contribute to its cofactor activity with APC [12,30]. EGF domains 2, 3, and 4 all contain the rare modified amino acid β-hydroxyasparagine [31], which provides high-affinity calcium-binding sites for these domains [32].

The final two laminin G-type (LamG) domains, spanning amino acid residues 258-434 and 443-625 [10], form the C-terminal sex hormone-binding globulin-like (SHBG) region of Protein S. This region contains three N-linked carbohydrate side chains. The second LamG domain contributes to APC-mediated cleavage of factors Va and VIIIa, while both LamG domains participate in binding to C4b-BP [33]. Although multiple residues have been implicated in C4b-BP binding, the precise binding interface remains unclear. The ProS SHBG-like domain, specifically its LamG1 subunit, binds the alpha isoform of tissue factor pathway inhibitor (TFPIα) and positions TFPIα’s second Kunitz-type inhibitory domain for optimal FXa inhibition. The LamG1–LamG2 region also engages the extracellular immunoglobulin (Ig) domains of Tyro3 or Mer to trigger receptor dimerization [19,34]. The modular domain architecture of ProS provides an integrated structural basis for its coordinated roles in coagulation, complement regulation, and TAM receptor signaling.

## 3. Anticoagulant Functions of ProS

ProS is unique among the plasma anticoagulants, as it promotes inhibition of all three canonical coagulation complexes: tissue factor (TF)/factor VIIa (FVIIa) (extrinsic tenase); factor VIIIa (FVIIIa)/factor IXa (FIXa) (intrinsic tenase); and factor Va (FVa)/FXa (prothrombinase). Not only that, ProS promotes the inhibition or degradation of five of the six individual proteins in these complexes, through a mixture of direct and indirect anticoagulant effects. The presence of APC-independent anticoagulant functions is further supported by recent work in zebrafish, which showed that, while knockouts of protein C and ProS both result in a prothrombotic state, the phenotypes are different [35], with intracardiac thrombosis observed in *ProS*^−/−^ zebrafish, while *ProC*^−/−^ fish experienced spontaneous venous thrombosis, as well as intracardiac thrombosis. These activities, along with their regulation by other plasma proteins, are discussed in this section.

### 3.1. APC Cofactor Function

The first and best-described anticoagulant function of ProS is as a cofactor for activated protein C (APC), the serine protease that degrades and inactivates FVa and FVIIIa. The ProS EGF1 and EGF2 domains interact with APC [12,36,37,38,39,40]. Through these interactions, ProS promotes the binding of APC to negatively charged phospholipids and subsequently increases the rate of degradation of FVa [2,41] and FVIIIa [42]. In addition to degrading FVa, APC also processes the precursor factor V (FV) [43], and APC-cleaved FV enhances the rate of degradation of both FVa and FVIIIa by the APC/ProS system [44,45]. The SHBG domain of ProS is necessary for these FV-dependent functions, while it is not required for FV-independent activity, suggesting that the SHBG domain interacts with a region of APC-cleaved FV that is not present in FVa [45]. Paradoxically, as APC activation is dependent on thrombin, low-dose dabigatran may promote a prothrombotic state by reducing APC generation [46]. This effect appears dependent on plasma ProS concentration. The APC cofactor function of ProS is also impaired by assembly of FVa into prothrombinase. The presence of FXa and prothrombin (the natural substrate for prothrombinase) protects FVa from degradation by APC/ProS [41,47,48].

As C4b-BP also binds the SHBG domain [49], one might anticipate that C4b-BP blocks APC cofactor activity, and indeed, Comp and colleagues described two patients with C4b-BP-bound ProS, but no detectable free ProS and no detectable APC cofactor activity in a plasma clotting assay [50]. Dahlbäck obtained similar results, observing a dose-dependent effect of exogenous C4b-BP added to C4b-BP-immunodepleted plasma or purified protein systems [19]. At saturating concentrations, C4b-BP almost completely inhibited the APC cofactor function of ProS, as measured by either plasma clotting time or residual FVa cofactor function. Subsequent studies showed that the effect of C4b-BP is more complex than simply inhibiting all activity. First, C4b-BP blocks the APC-mediated cleavage of FVa at Arg506, but the C4b-BP/ProS complex is capable of promoting cleavage at Arg306 [51]. Second, C4b-BP blocks the FV-dependent degradation of FVIIIa by ProS/APC, consistent with the requirement of the SHBG domain to interact with APC-cleaved FV; however, C4b-BP does not inhibit FV-independent degradation of FVIIIa in a purified system, suggesting that the effect of C4b-BP on ProS/APC function is substrate-dependent [52]. Third, the C4b-BP α chain also protects FVa from degradation by APC independent of ProS, suggesting that C4b-BP may directly interact with either APC or FVa [53]. The net result of this is that C4b-BP-bound ProS has greatly reduced anticoagulant activity, as evidenced by the prothrombotic phenotype in the patients described by Comp et al. [50].

### 3.2. TFPIα Cofactor Function

TFPI is an alternatively spliced protein with two primary vascular isoforms: TFPIα, a soluble protein containing three Kunitz-type protease inhibitor domains [54], and TFPIβ, which contains the first two Kunitz domains of TFPIα and is tethered to the cell membrane through a glycosylphosphatidylinositol anchor [54,55]. Both isoforms inhibit FXa [56,57] and the TF/FVIIa complex [56,58,59,60,61]. ProS binds the third Kunitz domain of TFPIα [62,63], and therefore does not interact with TFPIβ [64]. TFPIα binding is mediated by residues within the ProS LamG1 domain, with replacement of LamG1 by the homologous domain in growth arrest-specific 6 (Gas6) resulting in complete loss of cofactor function [63,65]. As LamG1 also mediates C4b-BP binding, exogenous C4b-BP blocks this interaction and completely reverses the TFPIα-dependent activity of ProS in plasma thrombin generation assays [66].

The exact affinity of the ProS/TFPIα interaction is unclear. In surface plasmon resonance experiments, this interaction is detectable but too weak to calculate binding parameters and is estimated to be in the low µM range, well above the physiologic concentration of either protein [62,63,67,68]. By contrast, the same studies reported that ProS cofactor activity is detectable in in vitro experiments using 1 nM TFPIα and low-to-mid nM concentrations of ProS, including measurements of FXa amidolytic activity and plasma thrombin generation. The reason for the discrepancy between measurements is unclear but may reflect the need for a phospholipid surface to promote binding. Regardless of the exact affinity, there is evidence that ProS and TFPIα may circulate as a complex in plasma, as plasma TFPIα is reduced in ProS-deficient patients and in plasma that has been immunodepleted of ProS, while TFPIα immunoprecipitates with ProS antibodies, and plasma TFPIα and ProS concentrations correlate in healthy donors [15,47,67,69]. ProS enhances the membrane-dependent inhibition of FXa, measured using a chromogenic peptidyl substrate [70,71]. Similar to the cofactor activity with APC, ProS localizes TFPIα to the negatively charged membrane surface, bringing it into proximity with membrane-bound FXa. Thus, ProS enhances the function of soluble forms of TFPI that contain the third Kunitz domain, such as plasma and platelet TFPIα, but has no effect on forms of TFPI that are otherwise associated with the membrane, including endothelial cell TFPIβ [62,64].

Just as FVa is resistant to degradation by APC and ProS when incorporated into prothrombinase, FXa is resistant to inhibition by TFPIα when FVa is present, even in the presence of ProS [47,72,73]. The observations that neither the APC/ProS nor the TFPIα/ProS system effectively inhibit prothrombin activation by prothrombinase suggest that this complex is particularly resilient and remains active for a prolonged period under physiologic conditions, as has been proposed [74]. However, those studies assessed either the APC/ProS or TFPIα/ProS system in isolation. These two anticoagulant systems function synergistically. TFPIα promotes the degradation of FVa by APC/ProS, while APC promotes the inhibition of FXa by TFPIα/ProS [47,75]. When all three anticoagulant proteins are present, thrombin generation is dramatically reduced [47,75,76].

**Figure 2:**
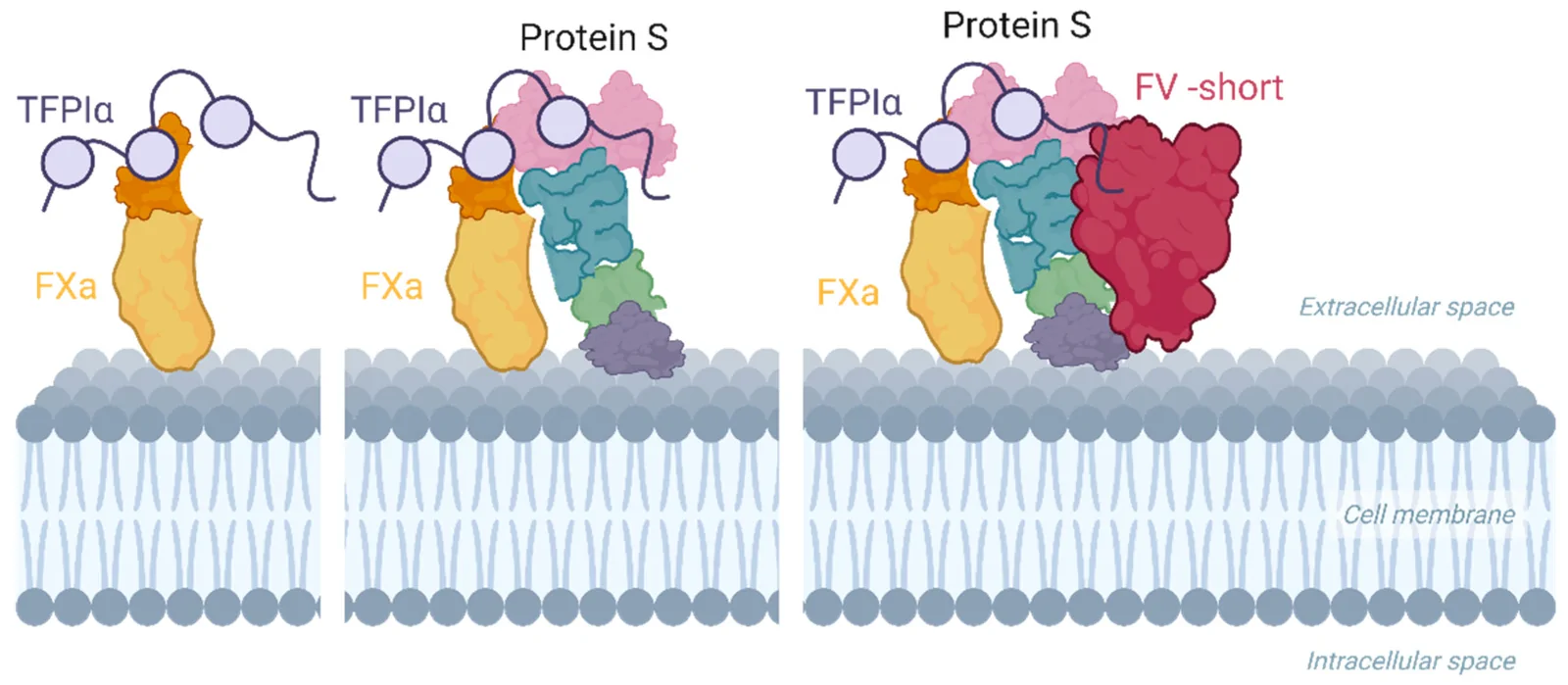
Inhibition of FXa. TFPIα inhibits FXa alone, when bound to Protein S, or when in a complex with Protein S and FV-short.

In addition to inhibiting FXa (Figure 2), TFPIα also inhibits the TF-FVIIa complex [56,58]. ProS promotes active-site inhibition of FVIIa by TFPIα, measured using a peptidyl substrate [77]. However, the effect of ProS on physiologic functions of TF/FVIIa (activation of FIX and FX) has been less clear, with some reports that ProS promotes inhibition of both TF/FVIIa functions by TFPIα by up to 10-15-fold [70,78]. This cofactor activity is dependent on phospholipids, suggesting that ProS acts by localizing TFPIα to the membrane surface, analogous to the mechanism of FXa inhibition. By contrast, others have reported that ProS does not alter the rate of inhibition of either TF/FVIIa function [71].

### 3.3. Direct Inhibition of FIXa

**Figure 3:**
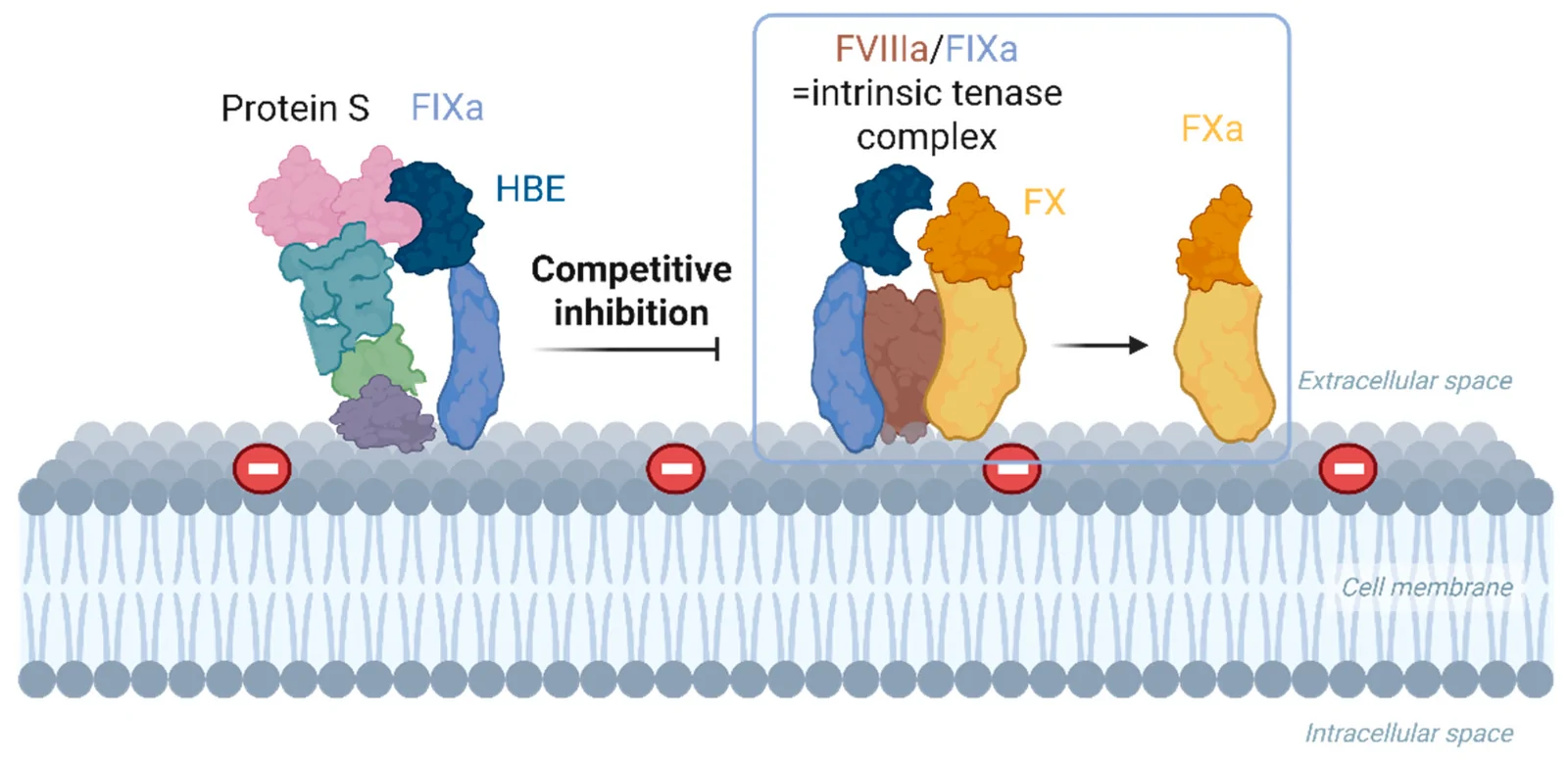
ProS-mediated inhibition of the intrinsic tenase complex. ProS binds the heparin-binding exosite (HBE) of FIXa via its C-terminal LamG1–LamG2 domains, competitively inhibiting Factor X activation by the FVIIIa/FIXa complex.

The cofactor functions of ProS described above are achieved through a similar mechanism, in which ProS localizes an effector molecule (APC or TFPIα) to the negatively charged membrane surface and, in doing so, enhances the activity of that effector molecule. This same mechanism is also observed with the anti-inflammatory functions of ProS described in Section 4 of this review. An interesting exception to the pattern, though, is the most recently described anticoagulant function: direct inhibition of FIXa. ProS was first shown to directly inhibit FIXa in 2012 [79]. ProS binds the heparin-binding site of FIXa [80] with a Kd of approximately 40nM [79], and this binding is mediated by the ProS LamG1 and LamG2 domains [81]. A recombinant protein consisting of the ProS LamG1-LamG2 domains binds FIXa with similar affinity to full-length ProS and with a similar effect [81]. Through this interaction, ProS competitively inhibits the activation of factor X by the intrinsic tenase complex, increasing the Km by approximately six-fold and making the reaction less efficient as a result [79] (Figure 3). This inhibitory function is observed in vitro and in vivo and is most apparent in conditions when coagulation is reliant on the intrinsic tenase complex, such as when initiated with low TF concentration. For example, in vitro ProS prolongs the activated partial thromboplastin time in a FIX-dependent manner, inhibits plasma thrombin generation initiated with 0.4 pM TF independent of TFPIα and APC, and inhibits activation of FX by the FVIIIa/FIXa complex in a purified system [79]. In vivo, FIXa mutated to prevent ProS binding (K132A/R170A) exhibited enhanced procoagulant activity in an electrolytic model when injected into a hemophilia B mouse, as compared to wild-type FIXa [80]. The published data are all consistent with the hypothesis that FIXa inhibition by ProS may be particularly relevant during pathologic conditions that are associated with intrinsic pathway activation and thrombotic risk, such as infection (recently reviewed in [82,83]), or under conditions of low-level TF exposure, as has been shown with TFPI [84]. However, while FIX Padua has been shown to be resistant to inhibition by ProS [85], no studies have yet directly demonstrated that this resistance contributes to a clinical outcome.

### 3.4. Modulation of ProS Anticoagulant Activity by FV-Short and VWF

Factor V (FV)-short, first described in 2013 [86], is an alternatively spliced isoform of FV that circulates bound to TFPIα. FV-short is a single-chain protein that contains a shortened activation peptide (B-domain). The FV-short B-domain contains a high-affinity binding site for TFPIα. TFPIα circulates in plasma with an average concentration of approximately 12.5 ng/mL (0.3 nM) [15]. FV circulates at an average of 20 nM in plasma [15,87], and FV-short is estimated to account for 1% of the plasma FV, or 0.2 nM [86]. As FV-short and TFPIα have similar concentrations in plasma and bind with a sub-nanomolar affinity [88], it has been hypothesized that virtually all TFPIα circulates in complex with FV-short, and vice versa. Consistent with this hypothesis, conditions that increase the circulating concentration of FV-short are also associated with elevated TFPIα and, as a result, bleeding risk [86,89,90,91]. As TFPIα has also been proposed to circulate in complex with ProS (see Section 3.2), a circulating trimolecular complex of ProS, TFPIα, and FV-short has also been proposed [92,93], and an AlphaFold model of this complex has been generated [94].

Multiple studies have delineated the effect of FV-short on the anticoagulant functions of TFPIα and ProS. Initial studies utilized other forms of FVa that retain a portion of the B-domain, including the TFPIα binding site, such as FXa-activated FVa or platelet-released FVa. Physiologic concentrations of TFPIα inhibit prothrombinase assembled with these forms of FVa [88], and this activity is not enhanced by ProS [64]. Subsequent studies employing recombinant FV-short showed that ProS potentiates TFPIα-mediated inhibition of both FXa amidolytic activity and thrombin generation by the prothrombinase complex. Notably, optimal inhibition requires the presence of both FV-short and ProS [91,95,96,97]. The synergistic functions of ProS, FV-short, and TFPIα were recently reviewed by Ahnstrom and colleagues [93].

Mathematical models have now been developed that incorporate the effect of FV-short based on published kinetic parameters. These models support the hypothesis that the described functions of the ProS/FV-short/TFPIα complex are sufficient to explain bleeding in conditions of elevated FV-short, with the models predicting a profound impact on thrombin generation [98]. In addition to pathologic consequences, FV-short and ProS also have pharmacologic implications. Andexanet alfa, a reversal agent for direct FXa inhibitors, binds and inhibits TFPIα, leading to thrombotic risk [99]. Interestingly, the anticoagulant function of TFPIα, in the presence of andexanet alfa, is restored by the addition of ProS and FV-short [100]. Specifically, in the presence of ProS and FV-short, andexanet alfa-bound TFPIα is able to effectively inhibit the TF/FVIIa complex. In contrast to TFPIα cofactor function, the effect of FV-short on the APC cofactor function of ProS is not yet clear. FV-short itself is protected from proteolysis by APC when in complex with TFPIα, though the effect of ProS has not been tested [101]. As APC-cleaved FV promotes the APC cofactor functions of ProS and retains the B-domain region that binds TFPIα [43], it is possible that FV-short works in a similar manner and promotes APC/ProS-mediated degradation of FVIIIa and/or FVa.

In 2025, we reported an additional protein that binds ProS and modulates its function, von Willebrand Factor (VWF) [102]. VWF unfolds when exposed to shear forces, binds ProS, and partially inhibits ProS anticoagulant activity. In a plasma thrombin generation assay, brief vortexing of the sample results in an approximately 10-15% decrease in ProS activity, which is entirely dependent on VWF. Similarly, in a microfluidic system, unfolded VWF localizes and clusters ProS on platelet thrombi, suggesting that VWF may alter the localization of ProS in vivo during clot formation. The exact binding sites for this interaction are unknown. However, VWF appears to bind within the LamG domains of ProS, as the interaction competes with TFPIα but not APC. Conversely, the binding site of ProS on VWF is shear-dependent and is likely located within the A1-A3 domain region of VWF. It is not yet known whether VWF-bound ProS has a physiologic function, but we did note that it is capable of interacting with Mer-Tyrosine Kinase (Mer-TK), a macrophage receptor which is discussed later in this review.

## 4. Anti-Inflammatory Functions of Protein S

In addition to down-regulating thrombotic responses, ProS also down-regulates inflammatory responses. This is achieved through two functions, both of which follow the extracellular scaffold mechanism described above. First, ProS promotes inhibition of the complement system by binding C4b-BP. Second, it promotes resolution of inflammation and efferocytosis of damaged cells by engaging TAM receptors.

### 4.1. C4b-BP Cofactor Function

**Figure 4:**
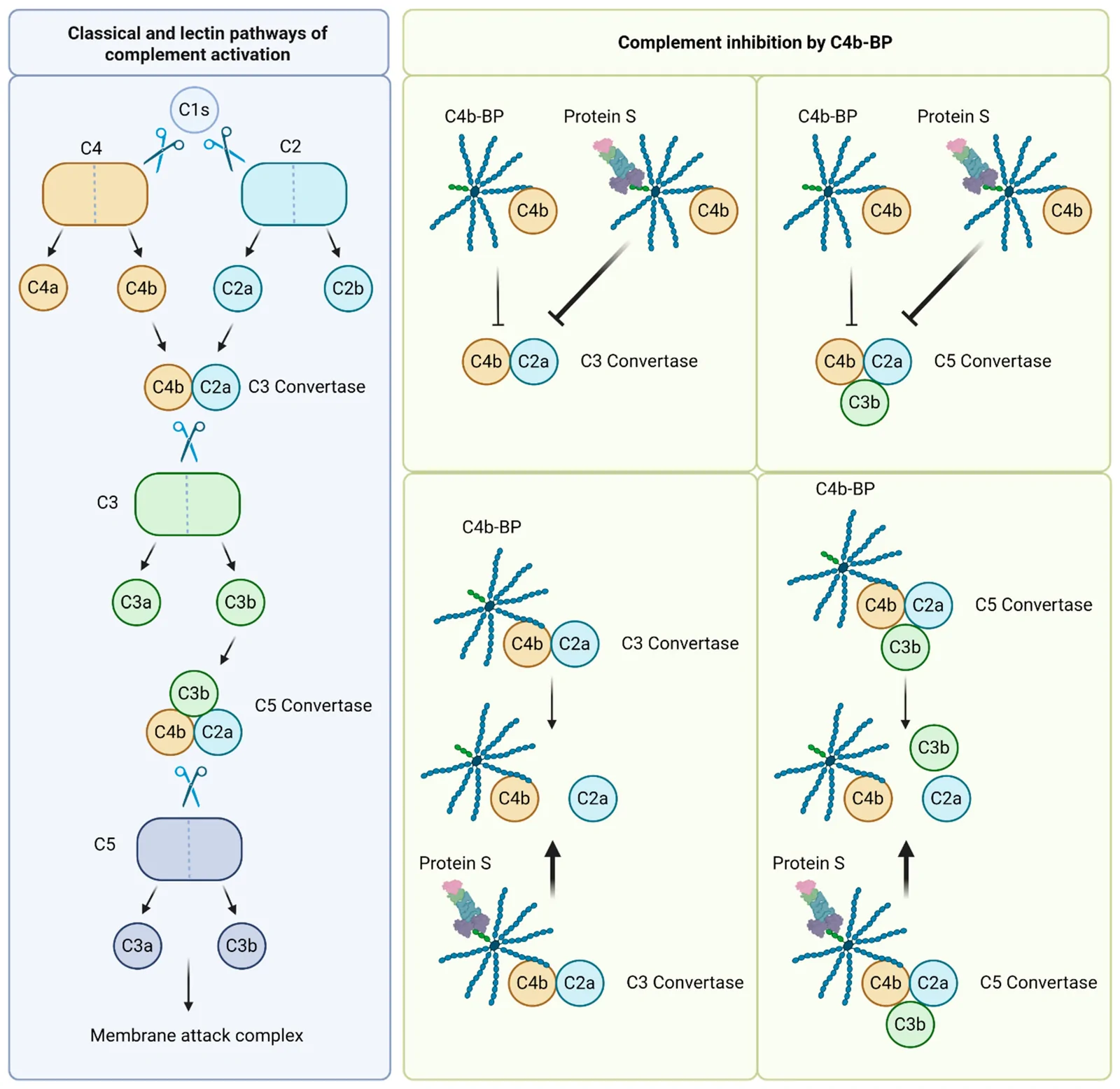
Regulation of the classical and lectin pathways of complement by C4b-BP and ProS. The C3 convertase is formed by the assembly of C4b and C2a. Its function is to cleave C3 into C3a and C3b. C3b then associates with C4b and C2a to form the C5 convertase. The formation of both C3 and C5 convertases can be inhibited by the binding of C4b-binding protein (C4b-BP) to C4b. C4b-BP can also bind to C4b within the C3 and C5 convertase complexes, promoting their dissociation. Protein S binding to C4b-BP promotes these inhibitory effects.

C4b-BP is a major soluble regulator of the classical and lectin pathways of complement activation, preventing excessive complement-mediated inflammation and tissue injury. C4b-BP, as its name suggests, binds complement component C4b, a component of the C3 convertase, which converts complement component C3 to C3a and C3b (reviewed in [103]). C3b assembles into the C5 convertase, which also includes C4b, and similarly promotes the conversion of complement component C5 to C5a and C5b. C5b is required to form the membrane attack complex, the terminal step of the complement pathway. By binding C4b, C4b-BP destabilizes the C3 and C5 convertases and promotes C4b degradation, indirectly limiting the formation of the membrane attack complex (Figure 4).

C4b-BP is a large plasma glycoprotein (~570 kDa) composed of seven α chains, which bind C4, and one β chain, which binds ProS with sub-nanomolar affinity [104]. Virtually all C4b-BP β chains are thought to circulate bound to ProS. However, not all C4b-BP contains a β chain. Three isoforms of C4b-BP are found in plasma: the predominant 7α-1β form (~160 µg/mL), the 6α-1β form, and the 7α form, which lacks the β chain and therefore does not bind ProS [18,105]. The concentrations of the minor C4b-BP isoforms have not been reported, but the total circulating concentration of C4b-BP is approximately 200 µg/mL [106]. These isoforms differ in their functional capacities, particularly in complement regulation and interactions with ProS [18,105]. As an acute-phase reactant, C4b-BP expression increases markedly during systemic responses to infection or tissue injury C4b-BP [105]. Hepatocytes up-regulate C4b-BP transcription under inflammatory cytokine signaling, and total plasma C4b-BP can rise up to four-fold. The predominant isoform, in this case, appears to be the β chain-deficient isoform [105]. The ProS Gla domain binds negatively charged phospholipid surfaces, such as those with exposed phosphatidylserine [12,25,26]. Through its interaction with the β chain [104], ProS is hypothesized to recruit C4b-BP to membrane sites where such phospholipids are exposed, such as apoptotic cells and circulating microparticles [107], and promote the binding of C4b-BP to those cells, thereby promoting inhibition of the C3 and C5 convertases [5,6]. Just as C4b-BP tends to increase during inflammation, free ProS tends to decrease under these same conditions, as we recently reviewed [108]. However, the concurrent increase in C4b-BP and decrease in free ProS may not be causally related, as free ProS deficiency can result from binding to VWF, which is also increased under the same inflammatory conditions [102].

### 4.2. TAM Receptor-Mediated Functions

Tyro3, Axl, and Mer-Tyrosine Kinase (Mer-TK) form the TAM receptor family and contribute to efferocytosis and control of inflammatory signaling, in part through their functional interplay with Toll-like receptors. TAM receptors are broadly distributed across many cell types, including macrophages, dendritic cells, monocytes, natural killer (NK) cells, B lymphocytes, regulatory T cells, CD4+ T cells, endothelial cells, epithelial cells, vascular smooth muscle cells, and neurons [109,110,111,112]. In addition, all three TAM receptors are expressed in platelets [113], and inhibition of Mer-TK reduces platelet activation and limits thrombosis [114,115]. TAM receptors are activated, and subsequent signaling is initiated by binding one of two recognized vitamin K-dependent ligands: ProS or its homolog Gas6 [116]. Engagement of Tyro3, Axl, or Mer-TK with their respective ligands enables these receptors to transmit context-dependent signals that restrain inflammation and promote the clearance of apoptotic cells (efferocytosis) [7]. Clearance of apoptotic cells is blocked by C4b-BP, suggesting that C4b-BP inhibits interaction with Mer-TK and/or Tyro3 [117].

Patterns of TAM receptor expression differ markedly among cell types, creating distinct signaling environments. Mer-TK is the dominant TAM receptor in macrophages, whereas Axl and Tyro3 are more typical in dendritic cells [118]. When combined with the unique binding preferences of TAM ligands, this distribution produces a highly selective signaling architecture. ProS interacts primarily with Tyro3 and Mer-TK, while Gas6 engages all three receptors but favors Axl [119]. This alignment of ligand specificity with receptor availability generates a finely tuned system in which ProS helps direct the anti-inflammatory and homeostatic functions of the TAM pathway (reviewed in [120]).

TAM receptor signaling inhibits innate inflammatory responses in dendritic cells and macrophages [121]. TAM signaling acts as a powerful brake on innate inflammatory activity, ensuring that responses to pathogens remain controlled and do not persist beyond what is necessary for host defense. When this regulatory pathway is compromised, the mechanisms that normally restrain inflammation begin to fail. As a result, insufficient TAM function is strongly associated with the development of chronic inflammatory states and contributes to the emergence of autoimmune disorders, where the immune system loses tolerance and mounts sustained, self-directed responses (reviewed in [110]).

**Figure 5:**
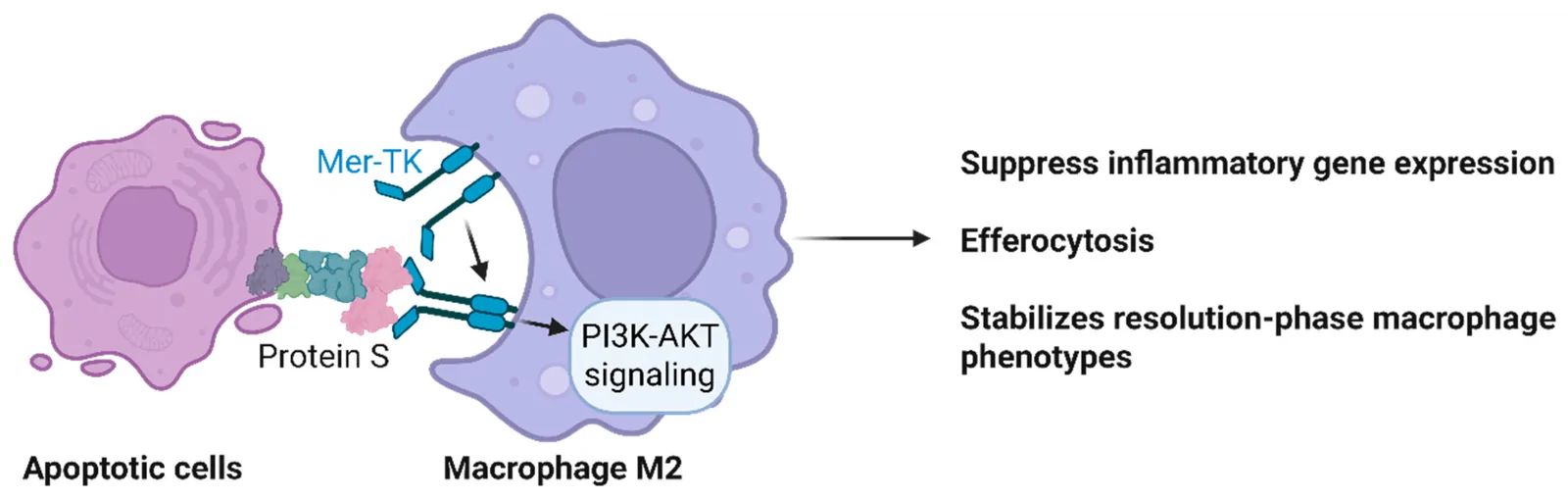
ProS-mediated Mer-TK activation and anti-inflammatory signaling. ProS acts as a molecular scaffold by binding apoptotic cell phosphatidylserine via its N-terminal Gla domain and phagocytic Mer-TK via its C-terminal SHBG-like domain. This localized engagement drives TAM receptor dimerization and autophosphorylation, activating downstream PI3K-AKT signaling to suppress inflammatory gene expression, enhance efferocytosis, and stabilize resolution-phase macrophage phenotypes.

Binding of ProS to Tyro3 and Mer-TK is mediated by the SHBG domain [122]. In addition, the interaction requires the ProS Gla domain, which binds phosphatidylserine exposed on apoptotic cells [107]. ProS dimerization on such surfaces is necessary for engaging TAM receptors [107,123]. Interaction with dimeric ProS promotes receptor dimerization and initiates autophosphorylation [123]. Once ProS activates Mer-TK or Tyro3, an intracellular signaling pathway is triggered along the PI3K-Akt axis [122]. PI3K recruitment and AKT phosphorylation generate a signaling environment that suppresses inflammatory gene expression, enhances efferocytosis, and stabilizes resolution-phase macrophage phenotypes (Figure 5) [23]. Recent studies show that macrophage-derived ProS is selectively increased during resolution, where it reinforces Mer-TK activity, sustains efficient efferocytosis, promotes the production of anti-inflammatory cytokines, and induces pro-resolving enzymatic pathways [23]. In the absence of ProS, macrophages are unable to sustain effective Mer-TK-driven efferocytosis and instead shift toward a pro-inflammatory activation state [23]. This suggests that genetic or acquired deficiency of ProS, or loss of ProS function, disrupts apoptotic cell clearance and augments inflammatory mediator release, positioning ProS as a central regulator of TAM-dependent resolution and a compelling candidate for therapeutic intervention in chronic inflammatory and fibrotic disease.

ProS plays a similar role in promoting the clearance of human immunodeficiency virus (HIV)-infected T-cells [124]. These cells expose phosphatidylserine and bind ProS, and the surface-associated ProS recruits macrophages through interaction with Mer-TK and promotes efferocytosis. ProS is consumed in this process, and this consumption is thought to contribute to the acquired ProS deficiency that persists in people living with HIV, even after treatment with antiretroviral therapy [76,125].

## 5. Dual Roles of Protein S in Tumor Progression and Suppression

As cancers are commonly associated with both inflammation and thrombosis, one might predict that the functions of ProS described above would be beneficial in reducing tumor burden. However, this is not always the case. In vitro, non-small-cell lung carcinoma (NSCLC) tumor cells secrete ProS, which promotes cell survival by binding and activating Tyro3 and Mer-TK on the cancer cells, triggering mitogen-activated protein kinase signaling to enhance cell proliferation, survival, and resistance to apoptosis that supports tumor growth and spread [126,127]. Simultaneously, activation of focal adhesion kinase signaling remodels the cytoskeleton, increasing cell migration, invasion, and metastatic dissemination [128]. ProS TAM signaling also stimulates angiogenesis and suppresses antitumor immunity, creating a microenvironment that supports tumor growth and spread [126]. A similar mechanism has been described in SCC-25 head and neck carcinoma cells, in which tumor-secreted ProS strongly stimulated Tyro3 phosphorylation, triggering downstream Erk kinase signaling (rather than Akt), protecting the cancer cells against apoptosis, and driving tumor cell survival [129]. These findings suggest that ProS may have an oncogenic role in some cancers.

By contrast, in other contexts, ProS can also act as a tumor suppressive factor by restraining inflammatory signals that fuel cancer progression. For example, ProS inhibits the development of some breast cancers by limiting invasion, migration, and extracellular matrix deposition, while its high expression is associated with increased immune cell infiltration and improved patient survival [130]. When ProS is absent, macrophages up-regulate production of tumor necrosis factor, interleukin-6, nitric oxide synthase-2, and interleukin-10, creating a protumor environment that drives epithelial–mesenchymal transition and activates extracellular signal regulated kinase, Akt, and STAT3 pathways, thereby enhancing tumor invasion and survival (Table 1) [131]. ProS deficiency in cancer patients can develop as a result of consumption, the inflammatory environment, or chemotherapeutic liver toxicity (reviewed in [132]). ProS also limits lung metastasis and suppresses TNF-α and IL-6 through Mer-TK kinase-dependent signaling [131]. In contrast, IL-10 regulation persists during Mer-TK kinase inhibition, suggesting a distinct regulatory mechanism. These findings highlight the role of ProS in controlling inflammatory responses and tumor invasiveness [131].

Furthermore, ProS functions as a tumor suppressor in bladder cancer by inhibiting the Akt/glycogen synthase kinase 3-beta (GSK3β)/β-catenin signaling pathway [133]. As TAM receptors signal through this pathway, these results are consistent with a mechanism in which ProS-mediated TAM receptor activation decreases Akt activation, allowing GSK3β to remain active. Active GSK3β promotes β-catenin degradation, preventing its accumulation in the nucleus and reducing the expression of genes involved in cell proliferation, migration, invasion, and angiogenesis [134,135].

As a result, bladder cancer cells exhibit slower growth and lower metastatic potential. These findings suggest that restoring ProS activity may represent a promising therapeutic strategy for suppressing bladder cancer progression [133]. In summary, host myeloid-derived ProS suppresses metastasis [131], whereas tumor-derived ProS promotes tumor progression by dampening antitumor immune responses, as evidenced by increased M1 polarization and enhanced antitumor immune responses following CRISPR-mediated deletion of ProS from B16F10 melanoma cells (Table 1) [136].

**Table 1:** Differential roles of host myeloid-derived and tumor-derived ProS in tumor progression and immune regulation.

Protein S Source	Experimental Model	Receptor	Biological Outcome	References
Tumor-derived ProS	Non-small-cell lung carcinoma (NSCLC)	Tyro3/Mer-TK	Enhances cell proliferation, survival, and resistance to apoptosis, supporting tumor growth and spread	[126,127]
Host myeloid-derived ProS	LLC lung carcinoma injected in myeloid-specific ProS knockout mice (Primary tumor growth and metastases)	Mer-TK	Primary tumor growth was unchanged, whereas lung metastasis increased following ProS deletion	[131]
Host myeloid-derived ProS	Bone marrow-derived macrophages (BMDMs) from myeloid-specific ProS knockout mice (Primary tumor growth and metastases)	Mer-TK	Limits pro-inflammatory macrophage responses	[131]
Tumor-derived ProS	Murine and human tumor models with Pros1-deficient tumor cells (Primary tumor growth and metastases)	Tyro3/Mer-TK	Increases macrophage M1 polarization and enhances antitumor immune responses.	[136]
Tumor-derived ProS	Human breast cancer cell line MDA-MB-231	Receptor Unknown	Limits invasion and migration; increased immune cell infiltration	[130]
Tumor-derived ProS	Bladder cancer (BLCA) cells	Receptor Unknown	Bladder cancer cells show slower growth and lower metastatic potential	[133]
Tumor-derived ProS	SCC-25 head and neck carcinoma cells	Tyro3	Protecting the cancer cells against apoptosis and enhancing tumor cell survival	[129]

## 6. Conclusions

**Figure 6:**
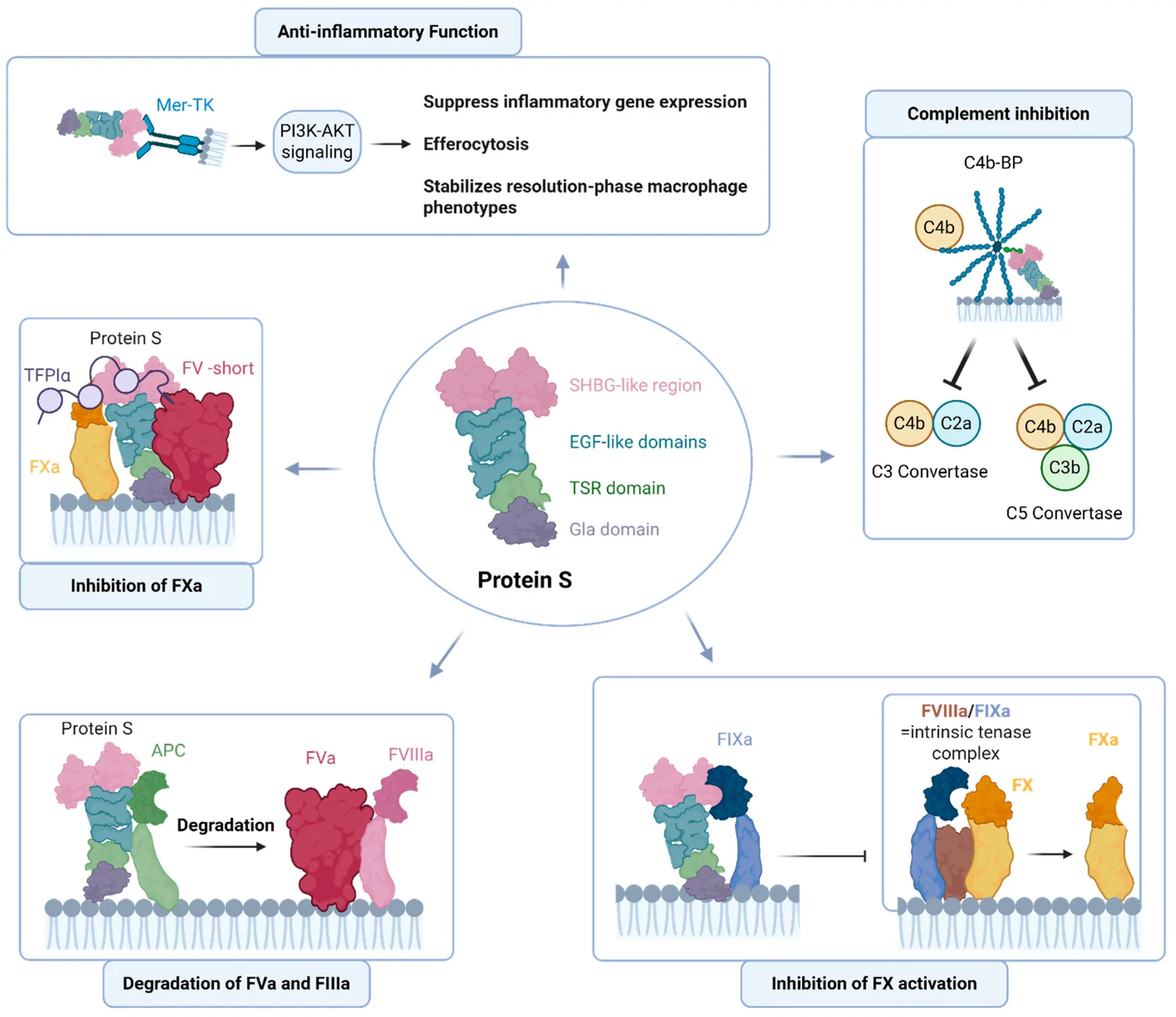
Protein S functions. Protein S consists of an N-terminal γ-carboxyglutamic acid (Gla) domain, a thrombin-sensitive region (TSR), four epidermal growth factor (EGF)-like domains, and a C-terminal sex hormone-binding globulin (SHBG)-like region. These domains contribute to the diverse functions of Protein S in the regulation of coagulation, complement activation, cell signaling, and inflammation.

ProS is a multifunctional protein that plays a central role in regulating coagulation and inflammation. Beyond its initially described cofactor activity with APC, ProS has a myriad of additional anticoagulant functions, regulating complement pathways, promoting efferocytosis, and mediating TAM receptor signaling, thereby linking hemostatic and inflammatory processes, both of which must be down-regulated as part of wound resolution. Most of the described functions of ProS largely share a common mechanism (Figure 6), in which ProS binds to phosphatidylserine-containing membranes and serves as a molecular scaffold that recruits effector molecules (APC, TFPIα, C4b-BP, TAM receptors). A notable exception to this model is FIXa inhibition. Rather than relying on a membrane-anchored assembly line, ProS directly binds FIXa and inhibits its proteolytic activity.

Despite the many discoveries since ProS was first described, much remains to be learned about this critical regulator of hemostasis and inflammation. As an example, knockout of the *ProS1* gene results in embryonic lethality in mice [17]. Are all functions of ProS critical for survival, or are some dispensable? If some functions are dispensable, can they be specifically targeted for therapeutic benefit? For example, it may be desirable to down-regulate the anticoagulant functions of ProS while retaining the ability to interact with C4b-BP and/or Mer-TK, or vice versa. Similarly, it may be possible to engineer ProS variants with impairment or enhancement of specific functions for therapeutic purposes. Acquired ProS deficiency is common in many clinical scenarios (reviewed in [108]), and it is plausible that restoring specific functions of ProS may provide therapeutic benefit, with those desirable functions varying by the particular condition. Finally, while we discussed numerous functions in this review, there are likely more functions still to be discovered. When we reported the interaction of ProS with VWF, we visualized plasma ProS by native gel electrophoresis and observed dozens of bands [102], suggesting that ProS may at least transiently interact with many other proteins in plasma. While some species of ProS were found to co-migrate with TFPIα, VWF, and FV, most remain unidentified. Future studies will continue to provide critical insights into the distinct roles that ProS plays across thrombosis, inflammation, and tumor development.

## Data Availability

No new data were created or analyzed in this study. Data sharing is not applicable to this article.

## Abbreviations

APC	Activated Protein C
C4b-BP	Complement component C4b-binding protein
EGF	Epidermal growth factor
Gla	γ-carboxyglutamic acid
Gas6	Growth arrest—specific 6
GSK3β	Glycogen synthase kinase 3-beta
Ig	Immunoglobulin
Mer-TK	Mer-Tyrosine Kinase
ProS	Protein S
SHBG	Sex hormone-binding globulin
TF	Tissue factor
TSR	Thrombin-sensitive region
VWF	Von Willebrand Factor
